# Intermuscular adipose tissue in obesity and related disorders: cellular origins, biological characteristics and regulatory mechanisms

**DOI:** 10.3389/fendo.2023.1280853

**Published:** 2023-10-18

**Authors:** Ting Zhang, Jun Li, Xi Li, Yanjun Liu

**Affiliations:** ^1^ Center of Obesity and Metabolic Diseases, Department of General Surgery, The Third People’s Hospital of Chengdu, Affiliated Hospital of Southwest Jiaotong University & The Second Affiliated Hospital of Chengdu, Chongqing Medical University, Chengdu, China; ^2^ Center of Gastrointestinal and Minimally Invasive Surgery, Department of General Surgery, The Third People’s Hospital of Chengdu, Affiliated Hospital of Southwest Jiaotong University & The Second Affiliated Hospital of Chengdu, Chongqing Medical University, Chengdu, China; ^3^ Medical Research Center, The Third People’s Hospital of Chengdu, Affiliated Hospital of Southwest Jiaotong University & The Second Affiliated Hospital of Chengdu, Chongqing Medical University, Chengdu, China; ^4^ Department of Orthopedics, The Third People’s Hospital of Chengdu, Affiliated Hospital of Southwest Jiaotong University & The Second Affiliated Hospital of Chengdu, Chongqing Medical University, Chengdu, China; ^5^ Institute of Life Sciences, Chongqing Medical University, Chongqing, China

**Keywords:** intermuscular adipose tissue, obesity, insulin resistance, intermuscular adipogenesis, therapeutic strategy

## Abstract

Intermuscular adipose tissue (IMAT) is a unique adipose depot interspersed between muscle fibers (myofibers) or muscle groups. Numerous studies have shown that IMAT is strongly associated with insulin resistance and muscular dysfunction in people with metabolic disease, such as obesity and type 2 diabetes. Moreover, IMAT aggravates obesity-related muscle metabolism disorders via secretory factors. Interestingly, researchers have discovered that intermuscular brown adipocytes in rodent models provide new hope for obesity treatment by acting on energy dissipation, which inspired researchers to explore the underlying regulation of IMAT formation. However, the molecular and cellular properties and regulatory processes of IMAT remain debated. Previous studies have suggested that muscle-derived stem/progenitor cells and other adipose tissue progenitors contribute to the development of IMAT. Adipocytes within IMAT exhibit features that are similar to either white adipocytes or uncoupling protein 1 (UCP1)-positive brown adipocytes. Additionally, given the heterogeneity of skeletal muscle, which comprises myofibers, satellite cells, and resident mesenchymal progenitors, it is plausible that interplay between these cellular components actively participate in the regulation of intermuscular adipogenesis. In this context, we review recent studies associated with IMAT to offer insights into the cellular origins, biological properties, and regulatory mechanisms of IMAT. Our aim is to provide novel ideas for the therapeutic strategy of IMAT and the development of new drugs targeting IMAT-related metabolic diseases.

## 1 Introduction

Obesity is associated with increased risks for diverse diseases, such as metabolic syndrome, type 2 diabetes, non-alcoholic fatty liver disease, and several cancers (1). Intermuscular adipose tissue (IMAT) is a unique adipose depot that expands between myofibers or adjacent muscle groups, which develops and progresses alongside the expansion of visceral and subcutaneous adipose tissue due to obesity (2). IMAT is distinct from the accumulation of lipids within myofibers, which is referred to as intramuscular lipids or intramyocellular lipids (3, 4). Imaging techniques have been increasingly used to noninvasively quantify IMAT, including computed tomography (CT) and magnetic resonance imaging (MRI) (5–7), and there is a good level agreement between IMAT assessment by MRI and histology (7). Several studies have suggested that IMAT poses a major threat to muscle metabolic disorders and physiological function, such as IR and muscle atrophy, in individuals with obesity, type 2 diabetes, and aging (5, 8, 9). Despite IMAT in the thigh being much less than subcutaneous adipose tissue (SCAT) in obese individuals, it is strongly correlated with IR (5). Additionally, IMAT in thigh muscle is independently associated with increased obesity-related heart failure risk after adjusting for cardiometabolic risk factors and other measurements of adiposity in humans (6). A separate study revealed that obesity-associated respiratory dysfunction in a mouse model was correlated with IMAT and collagen deposition within the diaphragm (10).

In recent years, several studies have suggested that IMAT adipocytes originate from muscle-resident stem/progenitor cells or other mesenchymal progenitors, resulting in the heterogeneity of intermuscular adipocytes with distinct metabolic characteristics (11, 12). For instance, human muscle-derived fibro/adipogenic progenitors (FAPs) *in vitro* give rise to white adipocytes that exhibit IR (13). Interestingly, one study reported the presence of brown progenitors in human skeletal muscle (14). Other researchers have demonstrated the existence of uncoupling protein 1 (UCP1)^+^ brown adipocytes within IMAT in mice, providing a therapeutic target for obesity by acting on energy dissipation (15). These findings indicate that there is still ongoing debate regarding the cellular origins and metabolic properties of IMAT adipocytes.

Therefore, within this context, we will review recent studies to explain the cellular origins of IMAT adipocytes and regulatory mechanisms involved in intermuscular adipogenesis. This review aims to provide new insights and potential targets for addressing IMAT-related conditions such as obesity, type 2 diabetes, and related disorders.

## 2 Metabolic characteristics of IMAT

**Figure 1:**
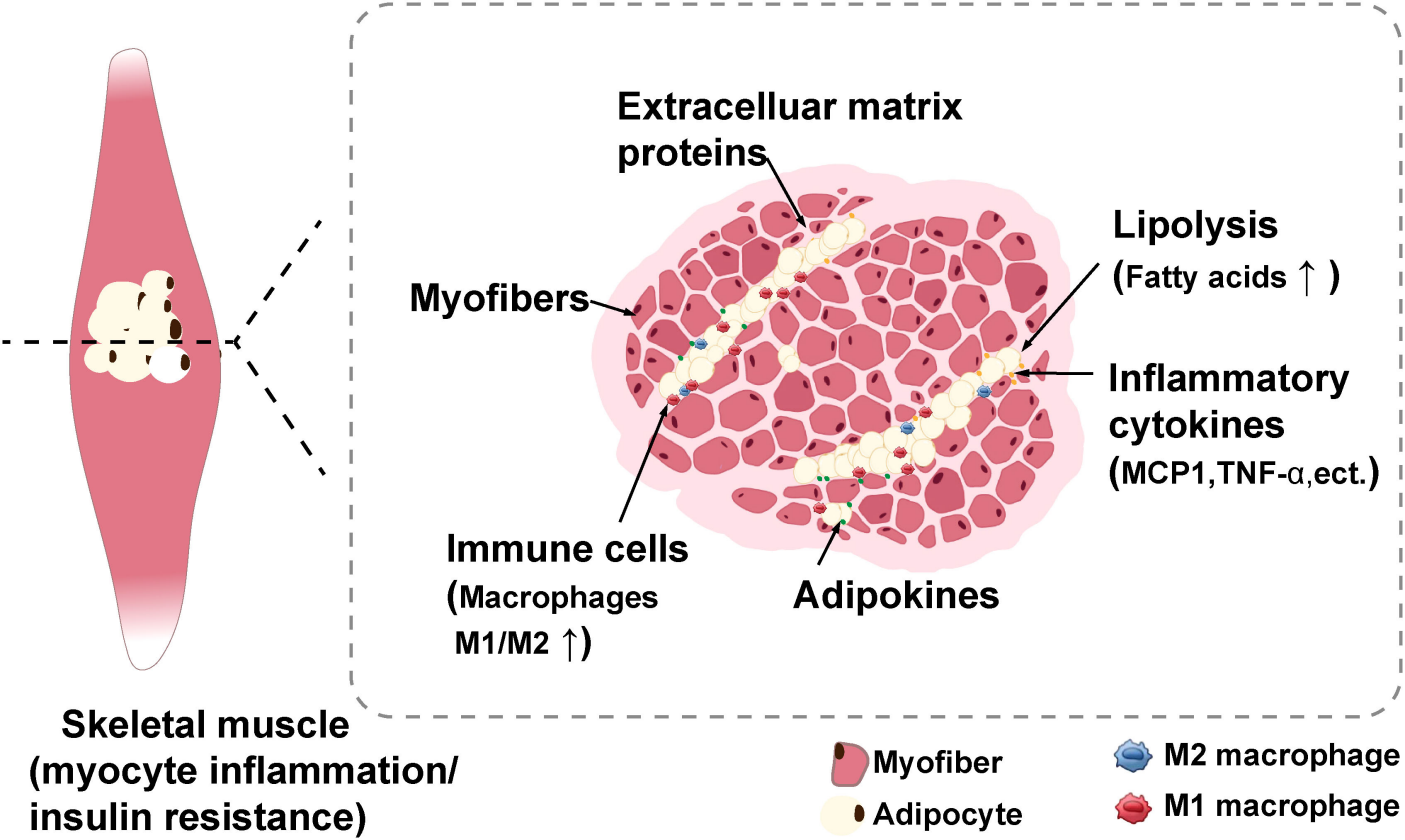
Schematic with potential mechanisms of myocyte inflammation and insulin resistance induced by IMAT (2). IMAT can synthesize and secrete numerous bioactive mediators such as inflammatory cytokines, adipokines, and extracellular matrix proteins to impart adverse effects, such as myocyte inflammation and insulin resistance. Moreover, these secretory inflammatory factors induces the recruitment of immune cells, particularly macrophages, which primarily infiltrate IMAT. IMAT, intermuscular adipose tissue. Created with BioRender.com.

Multiple studies have shown that IMAT is a robust predictor of metabolic abnormalities, such as IR, in both younger and older adults (5, 16). Sachs et al. conducted the first direct sampling and analysis of IMAT in humans and they found that the conditioned media for cultivating IMAT obtained from obese individuals reduced the insulin sensitivity of myotube from donors *in vitro* (17). Similar to other adipose tissue depots, IMAT synthesizes and secretes various bioactive mediators, such as inflammatory cytokines, and extracellular matrix proteins, which can lead to local inflammation or systemic inflammation, ultimately leading to decreased insulin sensitivity in humans (2, 8, 17–19). Furthermore, Sachs et al. discovered that IMAT contains macrophages proportional to insulin sensitivity, and macrophage cytokine secretion within IMAT such as monocyte chemotactic protein 1 (MCP1), is negatively related to insulin sensitivity (17). In obese humans, macrophage and T cells markers were upregulated in skeletal muscle compared with lean humans (20). In addition, macrophages, T cells, and other immune cells that respond to skeletal muscle inflammation are mainly situated in IMAT in diet induced-obese mice (20). These macrophages exhibit polarization toward the proinflammatory M1 phenotype (20, 21), exacerbating skeletal muscle IR and metabolic disorders (**Figure 1**).

Due to its negative impact on whole-body metabolism, IMAT adipocytes have been extensively studied *in vivo* and *in vitro*. Multiple studies have shown that adipocytes derived from muscle-resident mesenchymal progenitors in IMAT share similar characteristics with white adipocytes (9, 10, 13, 22). Liu et al. discovered that muscle-derived non-Pax3 myogenic lineage cells differentiate into white-like adipocytes *in vitro* (23). Girousse et al. demonstrated that the mobilization of CXCR4^+^ adipose stromal cells (ASCs) from SCAT toward skeletal muscle results in increased IMAT formation and subsequent impairment of glucose tolerance in mice (12).

Notably, different mouse strains exhibit differential susceptibility to diabetes and diet-induced obesity (15, 24). Almind et al. found that UCP1^+^ brown adipocytes within IMAT are more prevalent in obesity-resistant 129S6/SvEvTac (Sv129) mice than in C57BL/6 (B6) mice (15). Gorski et al. further found that FAPs provide a likely source for intramuscular adipocytes expressing UCP1 in obesity-resistant Sv129 mice (25). Schulz et al. reported that a subpopulation of adipogenic cells residing in murine skeletal muscle can differentiate *in vitro* into brown-like adipocytes when stimulated with bone morphogenetic protein 7 (BMP7) (26). Similarly, Crisan et al. demonstrated the presence of brown progenitors in human skeletal muscle that can differentiate into brown adipocytes *in vitro*, and they also found increased expression of UCP1 mRNA in adult human skeletal muscle, which was further enhanced by PPARγ agonist treatment (14). Additionally, Liu et al. uncovered that transplantation of brown adipose progenitors into mouse skeletal muscles leads to ectopic adipose tissue formation (27). Moreover, induced brown adipose progenitors can develop into brown adipocytes in mouse muscles, resulting in increased energy expenditure (27). Cai et al. demonstrated that transplanted brown adipose tissue (BAT) into the *quadriceps femoris* muscle of *ob/ob* mice significantly improved glucose homeostasis, alleviated obesity, and exhibited brown adipocyte characteristics (28), indicating that skeletal muscle could provide a microenvironment for brown adipogenesis.

In summary, despite the detrimental effects of IMAT on metabolism, the presence of brown adipocytes within IMAT offers a potential avenue for treating obesity (**Table 1**). The skeletal muscle microenvironment provides for maintaining intermuscular brown adipogenesis, offering a promising therapeutic strategy for IMAT-related morbid obesity and diabetes.

**Table 1:** The cellular origins and characteristics of IMAT adipocytes.

References	Cellular origins	Cellular marker	Study model	Cellular/metabolic characteristics
Asakura et al.(2001) (29)	Satellite cells	MyoD^-^Myf5^-^	Cell differentiation; *in vitro*	NA/NA
De Coppi et al.(2006) (30)	Human Satellite cells	CD44^+^CD56^+^HLA-ABC^+^CD3^-^CD4^-^CD45^-^CD31^-^	Cell differentiation (with Rosiglitazone); *in vitro*	White adipocyte/NA
Pasut et al.(2016) (31)	Satellite cells	Pax7^-^	Muscle regeneration; *in vivo*	PRDM16^+^Brown adipocytes/NA
Almind et al. (2007) (15)	Muscle resident cells	NA	Obesity (129S6/SvEvTac mice); *in vivo*	UCP1^+^adipocytes/positive
Crisan et al. (2008) (14)	Human muscle cells	CD45^-^CD56^-^CD146^-^CD34^+^	Muscle cells/mice/human (with Rosiglitazone); *in vitro* and *vivo*	UCP1^+^brown adipocytes/positive
Uezumi et al. (2010) (32)	Mesenchymal progenitor	CD31^-^CD45^-^SM/C-2.6^-^ PDGFRα^+^	Glycerol-induced fatty degeneration; *in vivo*	NA/negative
Laurens et al.(2010) (33)	Muscle-derived cells	CD34^+^CD56^-^	Human muscle cells/mice (glycerol-induced injury); *in vitro* and *vivo*	NA/negative
Schulz et al.(2011) (26)	Muscle resident progenitors (ScaPCs)	Sca-1^+^/CD45^-^/Mac1^-^	Human or obesity- mice muscle cells; *in vitro*	Like-brown adipocyte/positive
Uezumi et al.(2014) (34)	Mesenchymal progenitor	PDGFRα^+^	Human skeletal muscle disease; *in vivo*	NA/negative
Laurens et al. (2016) (22)	Human muscle stroma-vascular fraction (SVF)	CD56^-^CD15^+^	Cell differentiation; *in vitro*	White adipocyte/negative
Camps et al.(2020) (35)	Interstitial cell	Sca1^+^PDGFRα^+^CD142^-^	Muscular dystrophy; *in vivo*	NA/negative
Xu et al. (2021) (36)	Myeloid‐derived cells	Pdgfra^+^; Pdgfra^-^/Cd68^+^	Glycerol-induced injury; *in vivo*	NA/NA
Lu et al. (2022) (37)	Muscle progenitors	PDGFRβ^+^	Aging; *in vivo*	NA/negative
Joe et al. (2010) (11)	FAPs	Sca1^+^CD34^+^CD31^–^CD45^–^α7integrin^-^	Injury; *in vivo*	NA/positive
Arrighi et al.(2015) (13)	FAPs	PDGFRα^+^CD15^+^CD56^-^	Young and adult human muscle cells; *in vitro*	White adipocyte/negative
Buras et al.(2018) (10)	FAPs	CD31^-^CD45^-^Sca1^+^PDGFRα^+^	Obesity mice; *in vivo*	Some UCP1^+^; many UCP1^-^ cells/negative
Farup et al.(2021) (38)	FAPs	CD34^+^ CD90^+^ CD56^-^ CD31^-^CD45^-^	Type 2 diabetic patients muscle cells; *in vitro*	NA/negative
Hogarth et al. (2019) (39)	FAPs	Sca1^+^PDGFRα^+^	Muscular dystrophy/injury; *in vivo*	NA/negative
Girousse et al.(2019) (12)	Adipose Stromal Cells	CXCR4^+^	Diet-induced obesity; *in vivo*	White adipocyte/negative
Liu et al. (2019) (27)	Brown adipose progenitors(BAPCs)	NA	Transplantation with or without VEGF; *in vivo*	UCP1^+^ adipocytes/positive

NA, not available.

## 3 Cellular origins of IMAT

Based on previous studies, it has been indicated that muscle-resident stem/progenitor cells and other adipose tissue depot progenitors are potentially involved in the formation of adipocytes within IMAT (11, 12, 15, 35).

### 3.1 Muscle satellite cells

Multipotent SCs have the ability to differentiate into adipocytes, ultimately contributing to IMAT formation (29, 30, 40). In the absence of the myogenic transcription factor MyoD/Myf5, myoblasts derived from SCs undergo adipogenic or osteogenic differentiation (29). De Coppi et al. suggested that human SCs marked with CD44^+^CD56^+^HLA-ABC^+^ could differentiate into adipocytes when treated with rosiglitazone *in vitro* (30). Previous researches based on lineage tracing experiments have indicated that brown adipocytes can arise from Pax7/Myf5-expressing precursors in skeletal muscle (41–43). Seale et al. demonstrated that overexpression of *Prdm16* in myoblasts induces their differentiation into brown adipocytes *in vitro* (42). Yin and colleagues illustrated that the muscle-enriched microRNA-133 represses brown adipogenesis in skeletal muscle by targeting *Prdm16* mRNA in mice (43). Furthermore, Pasut et al. discovered that overexpression of the Notch1 intracellular domain (NICD1) in the *Pax7*-deficient SCs repressed both MyoD and microRNA-133, leading to brown adipocytes formation in regeneration muscle in mice (31). Thus, the regulation of satellite cell-derived brown adipocyte generation, targeting PRDM16 and microRNA-133, presents a crucial therapeutic target for combating obesity.

### 3.2 Fibro/adipogenic progenitors

Muscle-resident mesenchymal progenitors, specifically FAPs, are characterized by positive expression of platelet—derived growth factor receptor alpha (PDGFRα) and stem cell antigen-1 (Sca-1). These cells possess the ability to proliferate and differentiate into adipocytes (10, 11, 32, 34, 39). Camps et al. uncovered the presence of an interstitial CD142^−^ cell subpopulation within the Sca-1^+^PDGFRα^+^ population that undergoes adipogenic differentiation in skeletal muscle. They also discovered that the CD142^+^ cell population could inhibit adipogenesis by secreting growth differentiation factor 10 (GDF10) (35). Arrighi et al. confirmed that the PDGFRα^+^CD56^−^ muscle progenitors are identical to the CD56^−^CD15^+^ progenitors (13). Furthermore, they uncovered that adipocytes derived from FAPs exhibit a deficiency in UCP1 expression in both young and adult donors, and these adipocytes are insulin-resistant (13). Laurens et al. indicated that the CD56^−^CD15^+^ cell subpopulation isolated from the muscle of obese subjects differentiated into functional white adipocytes *in vitro*, which impaired insulin action and myofiber signaling (22). Collectively, the abovementioned studies suggest that FAPs and other PDGFRα^+^ progenitors have the potential for adipogenic differentiation.

### 3.3 Other muscle mesenchymal progenitors

Nonetheless, a subset of myeloid‐derived cells characterized by PDGFRα^−^CD68^+^ exhibited adipogenic potential (36). Lu et al. uncovered that PDGFRβ lineage cells from muscles undergo a fate transition, contributing to the infiltration of adipose and fibrotic tissues in old mice (37). Studies found that a population of muscle cells expressing the surface protein CD34 can differentiate into adipocytes *in vitro* (14, 33). Liu et al. showed that muscle-derived Pax3^−^ non-myogenic lineage cells differentiate into white-like adipocytes without UCP1 expression *in vitro* (23). These findings suggest that muscle-resident progenitor cells also have the potential for adipogenic differentiation under certain conditions.

### 3.4 Adipose stromal cells or progenitors

In addition to muscle-resident stem/progenitors, adipose stromal cells (ASCs) from SCAT can be released into circulation under the regulation of the chemokine CXCL12 and its receptor CXCR4 in mice (44). Girousse et al. demonstrated that CXCR4^+^ ASCs released from SCAT, upon exposure to a high-fat diet or CXCR4 antagonist directly promoted ectopic adipocyte formation in the muscle of mice, and subsequently impaired glucose tolerance in mice (12). In addition, one study has shown that there is a reservoir of brown progenitors, that is muscle cells expressing CD34, in human skeletal muscle, which can differentiate into brown adipocyte with a high level of UCP1 *in vitro* (14). Moreover, induced brown adipose progenitors can develop into brown adipocytes in the limb muscles of mice (27).

**Figure 2:**
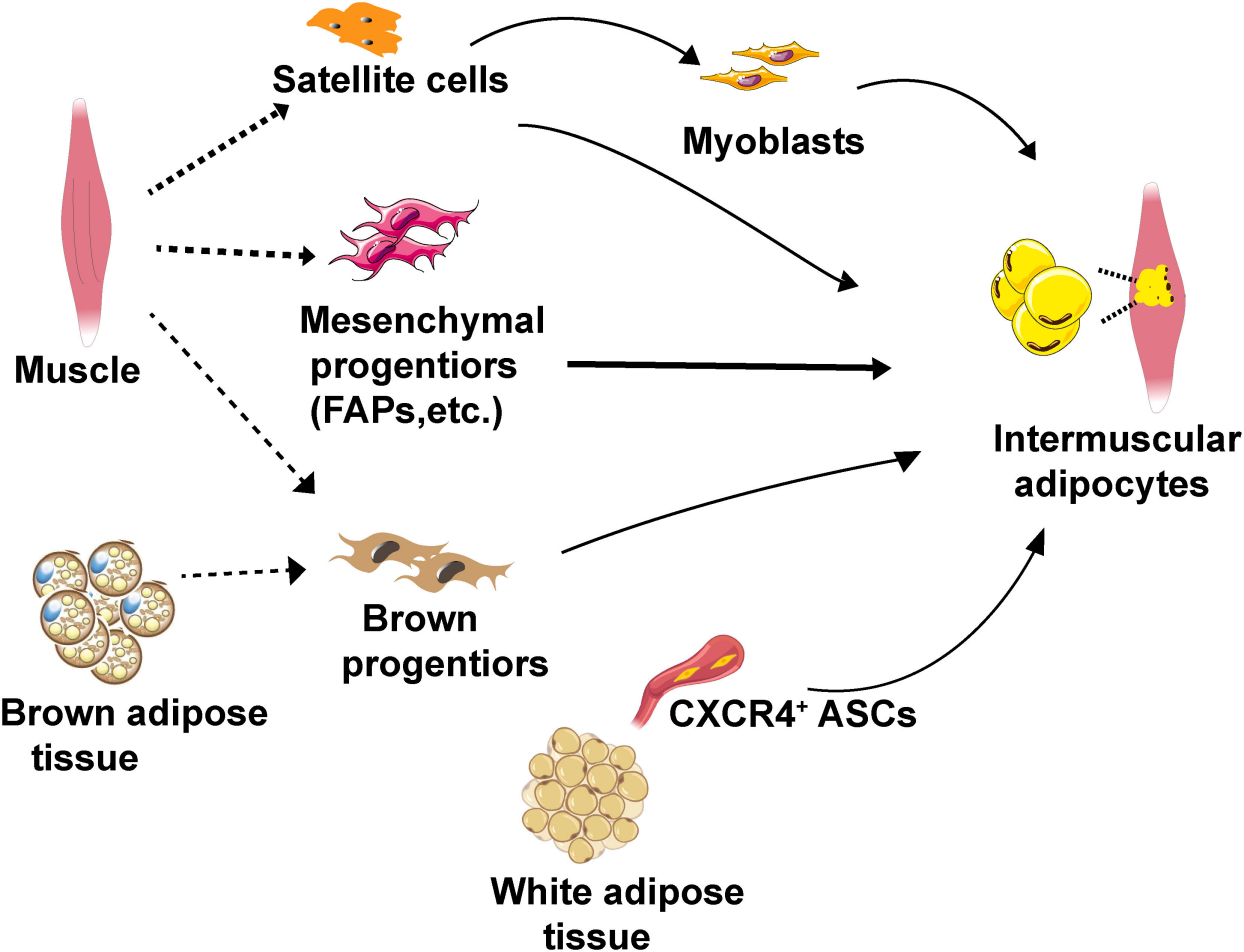
Schematic diagram of the cellular origins of IMAT adipocytes. IMAT adipocytes can potentially originate from muscle satellite cells, muscle-resident mesenchymal progenitors (specifically FAPs), CXCR4^+^ ASCs and brown progenitors. IMAT, intermuscular adipose tissue. FAPs, fibro/adipogenic progenitors. ASCs, adipose stromal cells. CXCR4, chemokine CXCL12 receptor.

Unlike classical adipose tissue depots, IMAT adipocytes exhibit heterogeneity, which may be attributed to their potential stem/progenitor cell origins (**Figure 2**). The characteristics of adipogenic progenitors of IMAT adipocytes play a crucial role in determining the metabolic traits of adipocytes in IMAT, thereby impacting whole-body metabolism (**Table 1**).

## 4 Regulatory mechanisms of intermuscular adipogenesis

### 4.1 Transcriptional regulators

Similar to classical adipose depots, intermuscular adipogenesis is regulated by a complex transcriptional cascade network that involves CCAAT enhancer-binding protein (C/EBP) family proteins, peroxisome proliferator-activated receptor gamma (PPARγ), sterol regulatory element-binding protein isoform 1c (SREBP1c), and fatty acid-binding protein (FABP4) (45, 46).

Apart from classical transcription regulators, Krüppel-like factor (KLF) family proteins play crucial roles in the differentiation of preadipocytes in livestock animals by interacting with C/EBPs and PPARγ. For instance, KLF4 inhibits the adipogenic differentiation of goat intermuscular preadipocytes *in vitro* by targeting C/EBPβ (47). KLF2 and KLF9 negatively regulate intermuscular adipogenesis (48, 49). KLF6 was the target gene of miR-22-3p and acted as an “on/off” switch in the differentiation of FAPs into adipocytes or myofibroblasts by regulating the matrix metallopeptidase 14 (MMP14) both *in vitro* and *in vivo (* 50 *).*

Fibroblast growth factors (FGFs) could also be potent regulators of adipogenesis in skeletal muscle. Basic FGF and FGF1 promote the differentiation of intramuscular adipocytes by regulating the expression of C/EBPα and PPARγ (51, 52). Sebastian et al. found that the conserved FGF2 increased IMAT formation in aged human skeletal muscle by inhibiting the adipogenic inhibitor SPARC (53). FGF21 negatively regulates the adipogenic differentiation of goat intermuscular preadipocytes *in vitro* by downregulating the expression of PPARγ and regulating the expression of numerous KLFs, including KLF3, 7, 9, 11, 14, and 16 (54).

### 4.2 Key signaling molecules

Previous studies have shown that multiple classical signaling pathways, such as the Hedgehog (Hh), Wnt, and Notch signaling pathways, can regulate IMAT formation in mouse models (55–57). Kopinke et al. demonstrated that Hh signals inhibit adipogenesis by regulating the expression of tissue inhibitors of metalloproteinase 3 (TIMP3) and MMP14 in a mouse model of injury-induced regeneration (55). Furthermore, other researchers uncovered a specific group of FAPs that were marked with glioma-associated oncogene homolog 1 (Gli1), which exhibited elevated Hh signaling and diminished adipogenic capability in a mouse model of muscle injury (58).

Wnt signals can act as a molecular switch controlling adipogenesis (59, 60). It has been suggested that Wnt10b inhibits adipogenesis by inhibiting PPARγ (60). Deceased Wnt10b signaling in myoblasts during aging induced adipose tissue infiltration in muscle (61). Similar results were observed in muscle SCs from obese Zucker rats (62). Reggio et al. identified Wnt5a as a noncanonical Wnt ligand that affects FAP adipogenesis by repressing PPARγ expression *in vitro* in a β-catenin-dependent manner (57). Brack and colleagues have shown that in a mouse model, the transition from Notch to Wnt signaling in myogenic progenitors is essential for effective muscle regeneration via glycogen synthase kinase 3 beta (GSK3β) (63). These findings potentially elucidate why, despite restoring the proliferative potential of Pax7^−/−^ SCs, NICD1 causes differentiation into brown adipose tissue (31). In addition to its effect on SC fate, Marinkovic et al. observed that myotubes inhibit FAP adipogenesis via Notch signaling *in vitro* (56). They further demonstrated that synergistic cooperation between Notch and inflammatory signals inhibits adipogenic differentiation in *mdx* FAPs (56).

In addition, Li et al. found that HMG20A exerts inhibitory effects on adipogenesis in porcine muscle SVFs and C3H10T1/2 cells through its interaction with lysine-specific demethylase 1 (LSD1) (64). Mozzetta et al. found that histone deacetylase inhibitors (HDACis) repressed the adipogenic potential of FAPs and enhanced the myogenic differentiation of SCs in young dystrophic mice but not in old *mdx* mice (65). Moreover, Wosczyna et al. uncovered that miR-206 repressed the adipogenic differentiation of FAPs by targeting Runx1 to limit intramuscular fatty degeneration in mice injured muscle (66).

To summarize, exploring innovative approaches to modulate the destiny of intermuscular preadipocytes or FAPs to inhibit intermuscular adipogenesis will be beneficial for controlling IMAT formation in pathological conditions.

### 4.3 The impact of the skeletal muscle microenvironment on IMAT formation

Skeletal muscle is a complex and plastic tissue, which includes myofibers, SCs, FAPs, immune cells, endothelial cells (67). The interactions between muscle-resident cells and paracrine signals from the microenvironment regulate the expansion and differentiation of adipogenic progenitors, thereby controlling the development of IMAT.

#### 4.3.1 The roles of myofibers in IMAT information

Previous studies demonstrated that the condition of myofibers affects IMAT accumulation (68, 69). Uezumi et al. found that myofibers strongly inhibit the adipogenic differentiation of PDGFRα^+^ cells in injured muscle in mice (32). Other studies showed variations in the adipogenic potential of preadipocytes in different muscles (23, 70). Liu et al. found that compared to the fast *extensor digitorum longus* (EDL) muscle, slow *soleus* (SOL) muscle contains more adipogenic progenitors in mice and these progenitors from SOL exhibits a higher propensity to form adipocytes *in vitro* (23), with the EDL muscle primarily consisting 80% type IIx and IIb fibers (glycolytic fibers) and the SOL muscle consisting 95% type I and IIa fibers (oxidative fibers) (71). In addition, Gu et al. showed that skeletal muscle-specific overexpression of PPARγ could significantly promote intramuscular fat deposition in the *longissimus dorsi* muscle but not in the *soleus* muscle in pigs (72). They further showed that overexpression of PPARγ in porcine muscle promotes the formation of slow oxidative fibers (72). These findings imply that myofiber type plays an important role in regulating intermuscular adipogenesis.

Studies have revealed that skeletal muscle-derived exosomes encapsulate the different myomiRs involved in local skeletal muscle tissue communication (73). They also found that the levels of these myomiRs within exosomes vary between skeletal muscles with different muscle fiber-type compositions (73). Chemello and colleagues showed differential expression profiles of microRNAs such as miR-206 and miR-499 between fast and slow myofibers (74). Wosczyna et al. uncovered that the adipogenic differentiation of FAPs was abrogated by miR-206 by repressing Runx1 translation in mice (66). Jiang et al. suggested that miR-499 hindered SCs adipogenic differentiation by reducing PRDM16 *in vitro* (75). Based on previous studies, we speculate that myofibers play a regulatory role in intermuscular adipogenesis.

#### 4.3.2 Myokines regulate intermuscular adipogenesis

Skeletal muscle, as a secretory organ, secretes bioactive myokines, including myostatin (MSTN), IL-15, irisin and IL-6, which likely exert both local (paracrine) and long-range (endocrine) effects. Studies have highlighted the potential roles of myokines in mediating tissue crosstalk and modulating the process of intermuscular adipogenesis.

MSTN, a member of the TGF-β superfamily, is a secreted protein that is specifically expressed in skeletal muscle, and is associated with myogenesis and adipogenesis in muscle development and regeneration (76, 77). Reisz-Porszasz et al. observed that transgenic mice overexpressing *Mstn* in skeletal muscle exhibited reduced muscle mass and increased fat mass (78). Lin et al. showed that increased muscle development in *Mstn* knockout (KO) mice may be associated with reduced adipogenesis (79). Artaza et al. demonstrated that recombinant MSTN promotes the differentiation of C3H10T (1/2) multipotent mesenchymal cells into the adipogenic lineage while inhibiting myogenesis *in vitro* (76). Additionally, Feldman and colleagues showed that MSTN can serve as a substitute for dexamethasone in inducing adipogenesis in C3H10T (1/2) cells but not in 3T3-L1 preadipocytes, which indicates that MSTN plays a role in promoting adipogenesis in the specific early stage (77). It should be noted that the adipocytes induced by MSTN in cell cultures and transgenic mice revealed the expression of markers associated with immature adipocytes, which exhibit favorable metabolic effects (77). However, inconsistent with previous findings, Liu et al. reported that the activated myostatin/SMAD4 signal promotes the expression of miR-124-3p, and inhibits adipogenesis by downregulating the expression of glucocorticoid receptor (GR) in porcine preadipocytes (80). Sun et al. suggested that MSTN inhibits intramuscular preadipocyte adipogenesis in a dose-dependent manner *in vitro* (81). Furthermore, they discovered that the culture supernatant from muscle tissue inhibits adipogenic differentiation of intermuscular preadipocytes *in vitro*. Zhang et al. indicated that MSTN inhibits the adipogenic differentiation of muscle SCs but not adipose-derived stem cells (82). Interestingly, Babcock and colleagues observed that the expression of MSTN and its receptor, activin receptor IIB (actRIIB), varied among different myofiber types in rat (83). They suggested that MSTN and actRIIB expression tends to be higher on IIx and IIb myofibers (I < IIa < IIx < IIb). Thus, depending on the context, MSTN can exhibit a dual role in the regulation of adipogenesis in skeletal muscle, either by inhibiting or promoting it.

IL-15 is a significant factor secreted by muscle fibers. It has been shown to inhibit the differentiation of porcine preadipocytes, specifically in the *longissimus dorsi* muscle, by suppressing the proliferation of preadipocytes in a dose-dependent manner *in vitro* (84). Another study revealed that the expression of IL-15 is negatively associated with fatty infiltration in injured human muscle (85). This study also found that IL-15 can stimulate the proliferation of FAPs and prevent the adipogenic differentiation of FAPs in injured muscle in mice (85). In addition, other myokines such as irisin (86, 87), IL-6 (88) and myonectin (89) also play an important role in the regulation of adipogenesis. These myokines are released by skeletal muscle in response to exercise and nutrients, suggesting that they may serve as potential therapeutic options for inhibiting IMAT accumulation.

### 4.4 Impact of trace elements on intermuscular adipogenesis

Dietary supplementation with trace elements, including vitamins and minerals, has the potential to regulate intermuscular adipogenesis by interacting with various regulatory factors.

#### 4.4.1 Vitamin A and retinoic acid signaling

Previous studies have shown that RA, an active metabolite of vitamin A, is a nutritional regulator of adipose tissue biology (90, 91). Berry et al. found that RA inhibits adipocyte differentiation *in vitro* by upregulating the expression of the adipogenesis inhibitors Pref-1, Sox9, and KLF2, and suppresses diet-induced obesity in mice (91). Zhao and colleagues demonstrated that RA effectively suppresses adipogenesis of FAPs in a dose-dependent manner *in vitro* (92). RA supplementation proves to be beneficial for obesity-impaired muscle regeneration by inhibiting both adipogenic and fibrotic differentiation of FAPs in mice (92). However, other researchers showed that neonatal supplementation with vitamin A leads to an increase in intramuscular fat levels without increasing overall fat levels (93). Their findings revealed that RA promotes angiogenesis and increases the number of intramuscular PDGFRα^+^ adipose progenitors *in vivo*, which subsequently leads to adipogenesis of intramuscular stromal vascular cells (SVCs) by activating VEGFA/VEGFR2 signaling (93). Therefore, during the early stage of IMAT development, changes in the muscle that impact extracellular matrix remodeling, along with the process of angiogenesis play a critical role (93, 94). Of note, it has also been shown that RA enhances adipocyte formation during the early stage but inhibits adipocyte hypertrophy at the terminal stage (93). While RA signaling inhibits white adipogenesis in murine cells through epigenetically inhibiting Zfp423 expression (95), it tends to downregulate ZFP423 in cattle SVCs, which aligns with the observation that RA downregulates the expression of adipogenic genes *C/EBPα* and *PPARγ* (93).

#### 4.4.2 Vitamin D

Studies have suggested a close relationship between vitamin D status and fat infiltration in muscle. Gilsanz et al. showed that serum 25-hydroxyvitamin D (25-OHD) levels were negatively correlated with the muscle fat percentage independent of body mass or subcutaneous and visceral fat measured by CT in 90 postpubertal females (96). In a clinical study on elderly individuals, IMAT in thigh muscles was significantly associated with both low vitamin D levels and poor physical performance (97), indicating that vitamin D may impact the deposition of IMAT. Ryan et al. reported that higher physiological concentrations of 1,25-OH_2_D_3_ inhibit IMAT formation (98). Supplementation with vitamin D alone or in combination with calcium can inhibit the expression of C-reactive protein (CRP), tumor necrosis factor (TNF)-α, and interleukin (IL)-6 (99), which partially explains the inhibition of IMAT formation in obese individuals. In addition, deficiency in vitamin D is associated with a decrease in the proportion and selective atrophy of type II (fast-twitch) fibers in elderly women (100), potentially altering the local microenvironment of muscles.

#### 4.4.3 Mineral factors: Copper (Cu), Zinc (Zn) and iron

Apart from vitamins, the mineral content also influences the biological processes of IMAT formation in animal models (101). Afonso et al. discovered through muscle transcriptome analysis that Cu and Zn may have a negative regulatory effect on intermuscular adipogenesis in groups of Nelore steers (101). Moreover, studies have suggested that an increased iron burden plays a pivotal role in the development of sarcopenia in rats (102). Additionally, transferrin receptor 1 (Tfr1)-mediated iron homeostasis regulates skeletal muscle development, regeneration and metabolism (103–105). Ding et al. revealed that how the specific deletion of *Tfr1* in SCs impairs skeletal muscle regeneration with activation of ferroptosis in mice (105), whereas SC-derived myofibers play a critical role in regulating intermuscular adipogenesis and maintaining the skeletal muscle microenvironment.

**Figure 3:**
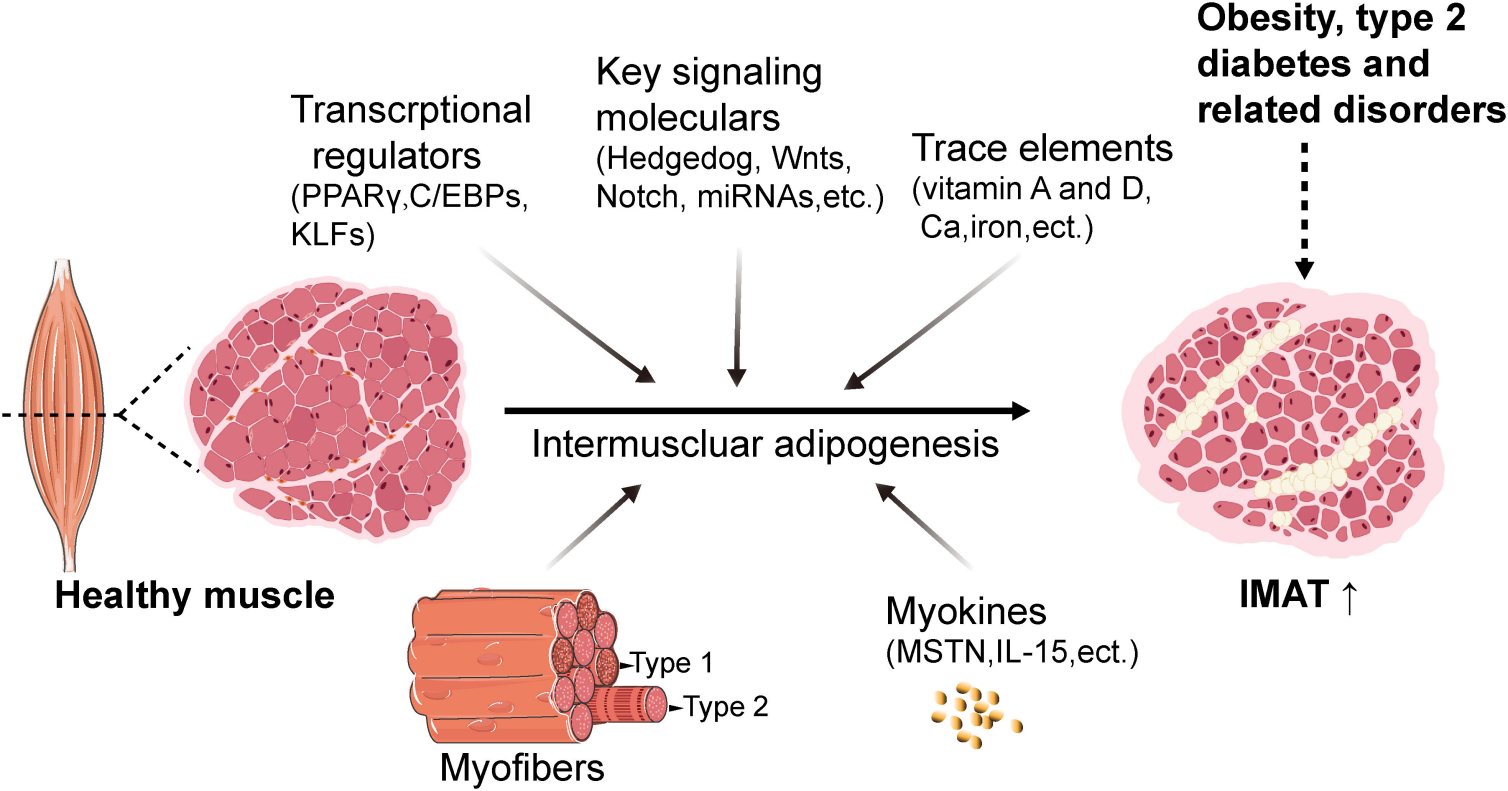
Schematic with proposed mechanisms of regulation of intermuscular adipogenesis. The regulation of intermuscular adipogenesis involves an intricate network cascade, which includes transcriptional regulators such as PPARγ, C/EBPs, and KLFs, as well as signaling molecules such as Wnt and Notch. Additionally, trace elements including vitamin A, vitamin D, calcium, and iron are involved. Furthermore, myofibers and myokines contribute to creating an essential microenvironment for intermuscular adipogenesis. IMAT, intermuscular adipose tissue. FAPs, fibro/adipogenic progenitors. C/EBPs, CCAAT enhancer-binding family proteins. PPARγ, peroxisome proliferator-activated receptor gamma. KLFs, Krüppel-like factor family proteins. MSTN, myostatin. Created with BioRender.com.

Currently, the regulatory mechanisms underlying IMAT formation are primarily investigated in domestic animal and rodent models. Accumulating evidence has suggested that the regulation of intermuscular adipogenesis involves an intricate network, involving the proliferation and differentiation of adipogenic precursors, the skeletal muscle microenvironment and nutritional regulators (**Figure 3**).

## 5 Potential interventions and therapies for IMAT: future research directions

Due to the detrimental effects of IMAT infiltration in skeletal muscle, clarifying the etiology, quantity and metabolic characteristics of its development is attracting increasing attention. However, the special anatomical location of IMAT limits its accessibility and the ability to conduct in-depth mechanistic studies. Earlier studies have investigated the origin and potential molecular regulatory mechanisms of IMAT adipocytes in livestock and rodent models, offering insights for clinical interventions to mitigate IMAT infiltration. We reviewed previous studies and found that skeletal muscle-resident mesenchymal progenitors, including PDGFRα^+^/Sca-1^+^ progenitors, and ASCs from other adipose depots serve as the primary source of IMAT, exhibiting characteristics similar to those of white adipocytes (12, 13, 22). Studies have demonstrated that inhibiting the proliferation and adipogenic differentiation of intramuscular FAPs can effectively impede the formation of intramuscular adipocytes. For instance, modulation of myokines, such as MSTN in the skeletal muscle microenvironment (80, 82, 83), and muscle fiber-derived miR-206, miR-499, can contribute to this inhibition (66, 75). In addition, researchers found that ASC trafficking is regulated by the CXCR4/CXCL12 axis, and pioglitazone intermittent treatment can prevent muscle ectopic fat deposition in high fat diet induced-obese mice (12).

Moreover, human skeletal muscle contains a reservoir of brown progenitors and provides a specialized microenvironment that supports intermuscular brown adipogenesis, which holds promise as a potential therapeutic target for obesity management (14, 27). However, although the expression of UCP1 is increased *in vivo* through PPARγ agonist treatment, the potential of adipocytes in the IMAT depot to serve as a fuel source for adjacent skeletal muscle remains unexplored in human subjects. Therefore, it will be a major challenge that how to facilitate intermuscular brown adipogenesis rather than white adipogenesis. Lineage tracing experiments have suggested that brown adipocytes in skeletal muscle can be derived from myogenic progenitors by modifying the expression of PRDM16 and miR-133 (42, 43). So it is necessary to investigate the potential molecular mechanisms of the transition from myogenic differentiation to brown adipogenic differentiation. In mouse models, the intermuscular brown adipocytes content was also affected by the species of mice, for example, more intermuscular brown adipocytes in obesity-resistant Sv129 mice than B6 mice (15, 25).

Additionally, in the context of obesity, the inflammatory response induces the recruitment of immune cells, primarily macrophages and T cells, which are predominantly located within the intermuscular adipose tissue. Moreover, macrophages undergo polarization into the proinflammatory M1 phenotype. Further research into the characteristics and potential molecular mechanisms of inflammatory cell infiltration in IMAT will also contribute to improving the management of metabolic disorders caused by IMAT.

## 6 Conclusions

Up to now, our understanding of the unique biology of IMAT, including its cellular, molecular, and biochemical mechanisms, has been enhanced primarily through IMAT tissue biopsy and related methodologies. However, knowledge concerning specific components of IMAT cell composition, secretion factors, and their influence on other metabolic tissues is still in its infancy. To fully uncover the impact of this unique adipose tissue on human health and diseases, additional comprehensive investigations into the quantity and biology of IMAT are crucial. While there is much work to be done, unraveling the mechanisms of IMAT infiltration will be an exciting area of future inquiry.

## Author contributions

TZ: Data curation, Methodology, Supervision, Writing – original draft, Writing – review & editing. JL: Supervision, Writing – review & editing, Data curation, Methodology, Writing – original draft. XL: Conceptualization, Supervision, Writing – review & editing. YL: Supervision, Writing – review & editing, Conceptualization.

## Glossary

**Table:**

IMAT	intermuscular adipose tissue
IR	insulin resistance
SCAT	subcutaneous adipose tissue
FAPs	fibro/adipogenic progenitors
UCP1	uncoupling protein 1
MCP1	monocyte chemotactic protein 1
ASC	adipose stromal cell
BMP7	bone morphogenetic protein 7
PPARγ	peroxisome proliferator-activated receptor gamma
SC	satellite cell
NICD1	intracellular domain of Notch1
PDGFRα	platelet-derived growth factor receptor alpha
Sca-1	stem cell antigen-1
C/EBP	CCAAT enhancer-binding protein
SREBP1c	sterol regulatory element-binding protein isoform 1c
FABP4	fatty acid-binding protein
KLF	Krüppel-like factor
FGF	fibroblast growth factors
Hh	Hedgehog
MMP14	matrix metallopeptidase 14
EDL	extensor digitorum longus
SOL	soleus
MSTN	myostatin
IL	interleukin
RA	retinoic acid
SVC	stromal vascular cell
Cu	Copper
Zn	Zinc
Tfr1	transferrin receptor 1.
